# Effects of Exercise-Based Programmes on Injury Incidence and Physical and Neuromuscular Outcomes in Futsal Players: A Systematic Review and Meta-Analysis of Randomized Controlled Trials

**DOI:** 10.3390/healthcare14162585

**Published:** 2026-08-17

**Authors:** Luis Cao-Bouza, Raquel Leirós-Rodríguez, Pablo Hernandez-Lucas, Esther Monge-Pereira

**Affiliations:** 1 Department of Functional Biology and Health Sciences, Faculty of Physiotherapy, University of Vigo, Campus A Xunqueira, 36005 Pontevedra, Spain; luis.bouza1@udc.es (L.C.-B.); esther.monge@uvigo.es (E.M.-P.); 2 SALBIS Research Group, Nursing and Physical Therapy Department, University of Leon, Astorga Ave., 24401 Ponferrada, Spain; rleir@unileon.es

**Keywords:** athletic injuries, injury prevention, exercise therapy, neuromuscular training, systematic review, sports, muscle strength

## Abstract

**Introduction:** Exercise programmes effectively prevent sports injuries and enhance neuromuscular parameters, but their impact on futsal performance remains understudied. **Objective**: The primary objective was to evaluate the effects of exercise-based programmes on injury outcomes in futsal players. The secondary objective was to assess their effects on physical and neuromuscular outcomes potentially associated with injury risk. **Material and Methods**: A search was carried out in PubMed, Scopus, SPORTDiscus, Web of Science, and PEDro databases. The methodological quality of the included studies was assessed using the PEDro scale, risk of bias using the RoB 2 tool, and certainty of evidence using the GRADE approach. A meta-analysis was performed using the Hedges’ g adjustment. **Results**: Fifteen reports corresponding to 12 unique studies and 405 randomized participants met the eligibility criteria. Two studies assessed direct injury outcomes. One reported significantly lower exposure-adjusted injury rates following the FIFA 11+ programme, whereas the other reported fewer injury events without a statistically significant between-group difference. Owing to differences in injury definitions and denominators, these findings were synthesized narratively. Random-effects meta-analyses showed significant effects favouring exercise-based programmes for muscle power (Hedges’ g = 0.616; 95% CI, 0.131 to 1.101; *p* = 0.013), muscle strength (g = 1.972; 95% CI, 0.214 to 3.731; *p* = 0.028), and flexibility (g = 0.569; 95% CI, 0.029 to 1.108; *p* = 0.039). The certainty of evidence was very low for injury outcomes and muscle strength and low for muscle power and flexibility. **Conclusions**: Exercise-based programmes may improve several physical and neuromuscular outcomes in futsal players. However, direct evidence regarding injury prevention is limited to two studies, and improvements in performance-related or biomechanical outcomes should not be interpreted as confirmation of a reduction in injury incidence.

## 1. Introduction

Futsal is a variant of football that is played in more than 100 countries and has approximately 12 million federated players worldwide [1]. During three consecutive FIFA Futsal World Cups, the injury incidence during matches was 195.6 injuries per 1000 player-hours (95% CI: 165.8–225.6) [2].

Major contributors to injury in futsal include previous injuries [3,4] and deficits in muscular strength, power, and agility [5,6]. In other football modalities, lack of flexibility, trunk stability, or reduced hip flexion range of motion have also been identified as risk factors [7,8,9,10,11].

Scientific evidence supports the use of injury prevention programmes, not only to reduce injury incidence, but also to improve athletic performance and minimize associated costs [3,6]. These programmes are often multidimensional and designed to address different neuromuscular parameters with the aim of mitigating modifiable risk factors [3,6]. One of the most researched prevention programmes is ‘FIFA 11+’, which has been shown to be effective in reducing injuries in 11-a-side football [12,13,14]. Although evidence of its impact on futsal is limited, early results suggest a decrease in injury incidence and improvements in neuromuscular performance [15,16].

Therefore, this systematic review and meta-analysis had two objectives. The primary objective was to evaluate the effectiveness of exercise-based programmes on injury incidence in futsal players, while the secondary objective was to assess their effects on physical and neuromuscular outcomes potentially associated with injury risk.

## 2. Methods

The review was conducted according to the methods specified in the registered protocol (PROSPERO: CRD42024549885), and reported in accordance with the PRISMA 2020 statement [17].

### 2.1. Eligibility Criteria

The eligibility criteria were structured using the PICOS framework as follows: P—population: futsal players regardless of gender, age, or level of play; I—intervention: exercise-based programmes designed either to prevent injuries or to improve physical or neuromuscular outcomes potentially associated with injury risk; C—control: other intervention, placebo, or no intervention; O—outcome: direct injury outcomes, including injury incidence, injury rate, or injury frequency, and physical or neuromuscular outcomes potentially associated with injury risk, including muscle strength, power, flexibility, range of motion (ROM), balance, proprioception, core function, and landing mechanics; S—study design: randomized controlled trials. Studies were excluded if they included participants with any type of injury or pathology. No restrictions were applied regarding language or year of publication. Only full-text reports of randomized controlled trials were eligible.

### 2.2. Search Strategy

A systematic literature review was conducted in January 2025 in the following databases: PubMed, Scopus, SPORTDiscus, Web of Science, and PEDro. The search strategy included a combination of the following MeSH terms: “Primary prevention”, “Exercise”, and “Exercise Therapy”. The following free terms were used: “futsal”, “indoor football”, “indoor soccer”, “prevention”, “exercise”, and “training”. Table S1 presents the search strategy according to the PICOS question.

### 2.3. Study Selection

The database search results were reviewed, and duplicates were removed. Then, two independent reviewers (L.C.-B. and P.H.-L.) evaluated the remaining articles to determine their suitability. In cases of disagreement between the reviewers, a third reviewer (E.M.-P) was consulted to make the final decision on whether to include or exclude a study from the analysis. This approach was used to ensure an impartial and fair evaluation of studies. After the initial data screening, the titles and abstracts were assessed according to the established inclusion criteria. The full texts of abstracts that met these criteria were obtained for further analysis. If the titles and abstracts did not provide sufficient information to determine their compliance with the inclusion criteria, their full texts were also retrieved. The selection of full-text articles was based on their adherence to the inclusion criteria, which was verified by two reviewers using a custom data extraction spreadsheet in Microsoft Excel. In cases of disagreement, the reviewers discussed the issue until they reached a consensus.

### 2.4. Data Extraction

Data extraction for further analysis was conducted by two reviewers (L.C.-B. and P.H.-L.). Descriptive information was extracted from the included studies, including publication details (author, year), sample sizes, age, gender, level of play, duration of the intervention, intervention groups, and outcome measurements. The characteristics of the intervention (start of treatment, session duration, number of sessions, and type of training) were also extracted. Data for each outcome to calculate effect sizes (mean and standard deviation) were obtained, and corresponding authors were contacted for additional data when necessary.

### 2.5. Quality Assessment, Risk-of-Bias Assessment, and Certainty of Evidence

Methodological quality was assessed using the Physiotherapy Evidence Database (PEDro) scale [18]. Risk of bias was assessed using the RoB 2 tool [19,20]. Although some overlap exists, PEDro was used to describe methodological quality, whereas RoB 2 provided an outcome-specific assessment of risk of bias. Certainty of evidence for each critical outcome was evaluated using GRADE [21]. All assessments were independently conducted by two reviewers (L.C.-B. and P.H.-L.), with disagreements resolved by consensus.

### 2.6. Data Analysis

Outcomes were grouped according to the underlying construct assessed. Lower-limb power referred to explosive performance measured through jump tests; muscle strength referred to lower-limb force or torque; flexibility referred to muscle extensibility; range of motion referred to joint angular excursion; core function included trunk endurance, activation, or motor control; balance referred to postural stability; proprioception referred to joint position sense; and landing mechanics included kinematic variables measured during landing tasks. Outcomes were quantitatively pooled only when they assessed the same underlying construct and had compatible directionality. Different instruments measuring the same construct were combined using standardized mean differences, whereas conceptually different outcomes were analyzed separately or narratively.

Standardized mean differences (SMDs) and their 95% confidence interval (CI) were calculated as the between-group difference in means divided by the pooled standard deviation (SD), using the Hedges’ g corrected effect sizes [22]. Hedges’ g was selected instead of Cohen’s d because it includes a correction for the upward bias associated with small sample sizes, which was relevant given the generally small trials included in this review [22]. While Cohen’s d and Hedges’ g are similar, we used Hedges’ g as it has better performance over Cohen’s d with inclusion of small samples [23]. When these data were not available in the study, they were requested via email to the authors. SMDs were interpreted using the following cut-off values: 0 to 0.2: very small; from 0.2 to 0.5: small; from 0.5 to 0.8: moderate; and 0.8 and above: strong [24]. Heterogeneity was measured through I^2^ statistics and explains how much of the variation between studies is due to heterogeneity rather than to chance. Values included between 0% and 40% may suggest “no important” heterogeneity, in a range from 30 to 60% indicates “moderate” levels, 50–90% may represent “substantial” heterogeneity, and 75–100% suggests “considerable” heterogeneity [25]. Given the anticipated clinical heterogeneity in the interventions and comparator conditions, random-effects models were used for all meta-analyses. The pooled estimates were interpreted as average effects across different exercise programmes and comparator conditions rather than as the effect of a single homogeneous treatment. Sensitivity analyses were performed to examine the influence of individual estimates on the pooled effects. Leave-one-study-out analyses were used when each study contributed one effect estimate, whereas leave-one-comparison-out analyses were used when multi-arm studies contributed more than one comparison. Formal subgroup analyses and meta-regression were not performed because the small number of studies resulted in insufficient studies within each potential subgroup. Analyses were performed with Comprehensive Meta-Analysis (CMA) V2 software (Biostat, Englewood, NJ, USA).

## 3. Results

### 3.1. Study Selection

**Figure 1:**
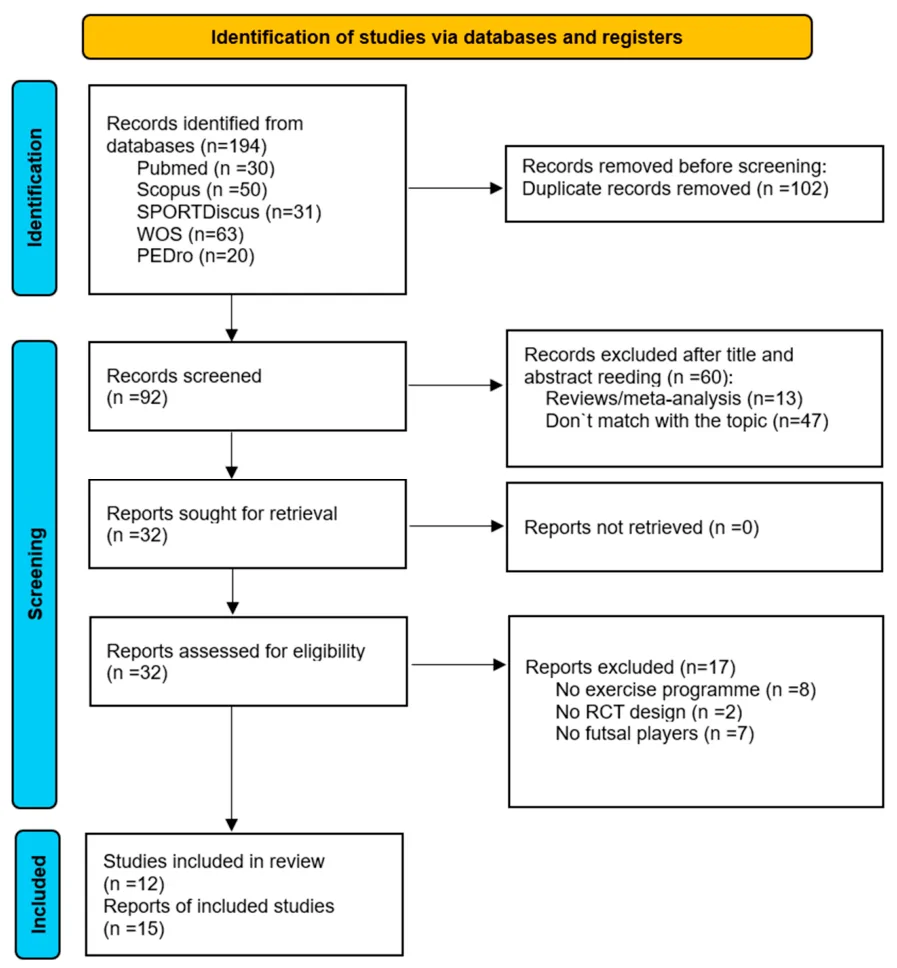
PRISMA flowchart.

The search identified 194 records. After removing 102 duplicates, 92 records were screened by title and abstract, of which 60 were excluded. The full texts of 32 reports were sought, retrieved, and assessed for eligibility. Seventeen reports were excluded: eight did not evaluate an exercise programme, two did not use a randomized controlled design, and seven did not include futsal players. The latter category included the two reports that evaluated soccer rather than futsal. Therefore, 15 reports corresponding to 12 unique studies met the eligibility criteria and were included in the review [26,27,28,29,30,31,32,33,34,35,36,37,38,39,40]. Figure 1 presents the PRISMA flow diagram. The corresponding authors of four reports were contacted to obtain additional data required for the analyses [27,28,31,37].

### 3.2. Methodological Quality and Risk-of-Bias Results

Methodological quality across the included articles after applying the PEDro scale (Table 1) showed two studies of excellent quality [29,36], five of good quality [26,27,28,38,40], seven of fair quality [30,31,32,33,34,35,37], and one study with a score of two points [39], and therefore of bad methodological quality. The most inconsistent criterion in the PEDro scale was the lack of blinding of subjects and programme administrators.

We also assessed the risk of bias of the different studies using the RoB 2 tool (Table 2). Among the studies that performed an intention-to-treat analysis, six papers had an unclear risk of bias [26,28,30,31,38], while three trials had a low risk of bias [29,36,40]. Only two RCTs had a high risk [27,37]. Risk of bias was most prevalent in the domains of sample randomization [26,27,28,30,31,37,38] and outcome reporting [26,27,28,30,31,37,38]. For trials that performed a per-protocol analysis, most had some risk of bias [32,33,34,35], while one study had a high risk of bias [39]. The domains most susceptible to risk of bias were sample randomization [32,33,34,35,39], data loss [32,33,34,35,39], and outcome assessment [32,33,34,39].

### 3.3. Certainty of Evidence

The GRADE assessment indicated very low-certainty evidence for direct injury outcomes and muscle strength, and low-certainty evidence for muscle power and flexibility (Table S2). Injury outcomes were downgraded for risk of bias, inconsistency, and imprecision; muscle power for risk of bias and substantial inconsistency; muscle strength for risk of bias, very serious inconsistency, and imprecision, particularly in the study by Reis et al.; and flexibility for risk of bias and imprecision. No outcome was downgraded for indirectness, while publication-bias analyses remained inconclusive because of the small number of studies.

### 3.4. Characteristics of the Sample

Across the 15 reports [26,27,28,29,30,31,32,33,34,35,36,37,38,39,40] corresponding to 12 unique studies, a total of 405 futsal players were included. The four reports by Lopes et al. [32,33,34,35] originated from the same cohort of 71 participants and were therefore counted only once. All participants were male, and the reported ages ranged from 15 to 34 years. Sex was explicitly reported in several studies. Ayala et al. [26] included 18 female professional futsal players, whereas Hamoongard et al. [30], the Lopes et al. trial [32,33,34,35], Pérez-Silvestre et al. [36], Reis et al. [38], and Saryono et al. [39] enrolled male players. Sex was not clearly reported in all remaining publications; therefore, no overall sex percentage was calculated.

Regarding the level of play, 10 trials were conducted on senior players [16,26,27,28,29,30,31,32,33,35,36,37,40] and two with junior players [38,39]. Among the senior studies, five involved professional players [26,27,30,31,37], while eight involved non-professional subjects [28,29,32,33,34,35,36,40] (Table 3).

### 3.5. Characteristics of Intervention Programmes

The mean number of sessions applied was 23, while most studies applied between 12 and 25 sessions [26,27,30,31,32,33,34,35,36,37,39,40]. The mean frequency was 2.3 sessions per week. The average duration of the sessions was 25 min, and most were applied as a warm-up, lasting between 15 and 25 min [26,32,33,34,35,37,38,39]. These types of programmes applied neuromuscular training with components of strength, plyometrics, agility, flexibility, and aerobic work [29,32,33,34,35,36,38,39,40]. One of the most widely used protocols was the FIFA 11+, used in five studies [32,33,34,35,38]. Four RCTs [29,30,31,40] applied programmes lasting between 30 and 60 min, divided into a warm-up phase, a main part, and a cool-down [29,30,31,40]. Two studies conducted interventions to improve core function [30,31]. Other studies applied other interventions such as the Pilates method [28], active stretching [26], rhythmic stabilization [27], or proprioceptive neuromuscular facilitation (PNF) [37].

Although the planned intervention dose was generally reported, the actual number of sessions completed was inconsistently documented. Lopes et al. [32,33,34,35] reported an average exposure of 1.9 ± 0.09 of the two planned sessions per week, while Reis et al. [38] reported 1.8 ± 0.1 of the two planned sessions per week. The remaining studies generally reported programme completion or participant attrition without providing individual attendance data, preventing a pooled estimate of adherence.

The interventions were supervised by physiotherapists in two of the studies [27,36]. The rest of the training programmes were administered by the team’s own coaches [26,30,31,32,33,34,35,36,39,40] or sport science professionals external to the coaching staff [28]. In some cases, a physiotherapist instructed these coaches on how to deliver the intervention [36]. Three of the trials did not specify which type of professional supervised the intervention [29,37,38]. Specific characteristics of the interventions carried out in each of the included studies are given in Table S3.

### 3.6. Effects on Injury Incidence

Two studies assessed direct injury outcomes among futsal players [27,35], although they used different injury definitions and surveillance methods. Lopes et al. [35] used a time-loss injury framework, recorded training and match exposure, and classified injuries according to onset, anatomical location, context, and severity. During the regular season, 24 injuries occurred in the FIFA 11+ group and 34 in the control group, corresponding to overall injury rates of 6.5 and 11.6 injuries per 1000 player-hours, respectively. The absolute rate difference was −5.1 injuries per 1000 player-hours (95% CI: −9.1 to −1.1; *p* = 0.014). The intervention group also showed lower rates of acute injuries (5.7 vs. 11.2 injuries per 1000 player-hours; rate difference: −5.5, 95% CI: −9.4 to −1.6; *p* = 0.007) and lower-limb injuries (4.4 vs. 8.7 injuries per 1000 player-hours; rate difference: −4.2, 95% CI: −8.1 to −0.4; *p* = 0.032).

Bello et al. [27] did not provide a formal operational definition of injury or record exposure time. The study reported clinically identified lower-limb muscular and ankle joint lesions during the four-month follow-up, with five events in the rhythmic stabilization group and nine in the passive stretching group; the between-group difference was not statistically significant (*p* = 0.096). As the studies differed in their injury definitions and denominators, and Bello et al. did not report exposure time or the number of players who sustained at least one injury, a comparable risk ratio or incidence rate ratio could not be calculated. Therefore, the findings were synthesized narratively rather than quantitatively pooled.

### 3.7. Effects on Muscle Power

Five studies, providing six comparisons, were included in the meta-analysis of muscle power [29,33,36,37,38]. The random-effects model showed a statistically significant moderate effect favouring the intervention groups (Hedges’ *g* = 0.616; 95% CI, 0.131 to 1.101; *p* = 0.013) (Supplementary Materials Figure S1). Substantial heterogeneity was observed (Q = 11.281; *p* = 0.046; I^2^ = 55.68%; τ^2^ = 0.194). The leave-one-comparison-out analysis produced effect estimates ranging from *g* = 0.457 to 0.787. Although all estimates favoured the intervention, statistical significance was lost after excluding Lopes et al. or Reis et al., indicating limited robustness. No evidence of small-study effects was identified using Begg and Mazumdar’s test (Kendall’s τ = −0.200; *p* = 0.573) or Egger’s regression test (intercept = −0.571; *p* = 0.821). Duval and Tweedie’s trim-and-fill procedure did not impute any missing comparisons.

### 3.8. Effects on Muscle Strength

Four studies were included in the meta-analysis of muscle strength [29,34,38,39]. The random-effects model showed a statistically significant large effect favouring the intervention groups (Hedges’ *g* = 1.972; 95% CI, 0.214 to 3.731; *p* = 0.028) (Supplementary Materials Figure S2). Considerable heterogeneity was observed (Q = 68.874; *p* < 0.001; I^2^ = 95.64%; τ^2^ = 2.886). The leave-one-study-out analysis produced effect estimates ranging from *g* = 0.216 to 3.096. Statistical significance was lost after excluding Lopes et al. or Reis et al.; notably, removal of Reis et al. reduced the pooled effect to *g* = 0.216 (95% CI, −0.412 to 0.845; *p* = 0.500), indicating that the overall estimate was not robust and was strongly influenced by this study. No statistically significant evidence of small-study effects was identified using Begg and Mazumdar’s test (Kendall’s τ = 0.667; *p* = 0.174) or Egger’s regression test (intercept = 9.118; 95% CI, −4.042 to 22.278; *p* = 0.097). However, Duval and Tweedie’s trim-and-fill procedure imputed one potentially missing study, reducing the adjusted random-effects estimate to *g* = 0.216 (95% CI, −1.926 to 2.357).

### 3.9. Effects on Muscle Flexibility

Three studies, providing four comparisons, were included in the meta-analysis of muscle flexibility [28,33,37]. The random-effects model showed a statistically significant moderate effect favouring the intervention groups (Hedges’ g = 0.569; 95% CI, 0.029 to 1.108; *p* = 0.039) (Supplementary Materials Figure S3). Low-to-moderate heterogeneity was observed (Q = 4.683; *p* = 0.196; I^2^ = 35.95%; τ^2^ = 0.111). The leave-one-comparison-out analysis produced effect estimates ranging from g = 0.304 to 0.878. Statistical significance was lost after excluding Bertolla et al. or either comparison from Portilla-Dorado et al., indicating limited robustness. No statistically significant evidence of small-study effects was identified using Begg and Mazumdar’s test (Kendall’s τ = 0.333; *p* = 0.497) or Egger’s regression test (intercept = 2.555; 95% CI, −2.375 to 7.485; *p* = 0.155). Duval and Tweedie’s trim-and-fill procedure imputed two potentially missing comparisons, reducing the adjusted random-effects estimate to g = 0.266 (95% CI, −0.293 to 0.826).

### 3.10. Effects on ROM in Hip Flexion

Two studies assessed the effects of their interventions on hip flexion ROM after applying exercise-based programmes [26,27]. Both achieved significant improvements in this variable [26,27].

### 3.11. Effects on the Function of the Core Musculature

Two studies evaluated the function of the core musculature after the application of different training programmes [30,31]. The studies observed significant increases in the function and motor control of the deep stabilizing core muscles when an exercise programme was applied [30,31].

### 3.12. Effects on Balance

Two studies examined the effects on balance [32,38]. Lopes et al. [32] found no significant short- or long-term between-group differences in static balance, assessed using a single-leg stance test, or dynamic balance, assessed using the Y-balance test, following the FIFA 11+ programme. Reis et al. [38] reported an improvement in unilateral balance of the non-dominant limb following the FIFA 11+ programme, whereas no significant between-group difference was observed for balance on the dominant limb.

### 3.13. Effects on Proprioception

Two studies assessed knee proprioception using joint position sense tests [32,36]. Lopes et al. [32] found no significant short- or long-term changes in knee joint position sense following the FIFA 11+ programme. Pérez-Silvestre et al. [36] reported significantly lower, and therefore better, absolute angular error and variable angular error values in the intervention group than in the control group at the 10-week follow-up. No significant between-group difference was observed for relative angular error, and no significant differences were found at 6 weeks.

## 4. Discussion

The aim of this systematic review with meta-analysis was to gather the available evidence on various exercise programmes in futsal players, and evaluate their impact on injury incidence and physical and neuromuscular outcomes potentially associated with injury risk. The results obtained suggest that the implementation of multidimensional exercise programmes has positive effects on several physical and neuromuscular outcomes, although direct evidence regarding injury prevention remains limited.

The programmes showed potentially beneficial effects on injury outcomes, although the available evidence was limited and could not be quantitatively pooled [27,35]. This is consistent with the positive results obtained after the implementation of this type of programme in 11-a-side football [12,13,14]. Independently, Bello et al. [27] found fewer injuries when implementing a rhythmic stabilization programme in professional futsal players, although the differences with the control group that performed passive stretching were not significant [27]. This could be because static stretching is effective in reducing muscle stiffness, which is associated with an increased risk of injury in high-intensity sports such as futsal [41].

The interventions applied succeeded in increasing muscle power, with a moderate effect size [29,33,36,37,38]. The incorporation of exercises to improve muscle power in the lower limbs is common in prevention programmes [42,43]. In the same vein, other studies have shown that exercise programmes are effective in improving muscle power in futsal and team sports [44,45]. It is important to highlight that this variable is fundamental for injury prevention in this sport, because it is considered one of the main modifiable risk factors [5,6].

Strength also improved significantly and with a large effect size [29,34,38,39]. In line with previous reviews [44,46,47], such programmes could positively affect injury incidence given the relationship between muscle strength deficits and increased risk of musculoskeletal injuries [7,11,29].

In terms of flexibility, studies also managed to improve flexibility, albeit with a moderate effect size [28,33,37]. While there is support for the idea that flexibility deficits increase the risk of injury in team sports [48,49], the relationship between flexibility and injury prevention remains controversial in some contexts. Indeed, it is not clear that an exclusively flexibility-based approach is sufficient to reduce injury incidence [5,42]. However, programmes that include flexibility components, along with other multidimensional approaches, have been shown to be effective in reducing muscle injuries [43,50]. Improving hip flexion ROM may also play an important role in reducing injury risk, as suggested by some studies [9,51]. Therefore, exercise programmes aimed at improving hip flexion ROM could provide benefits in terms of injury prevention [9]. In this line, two studies analyzed in the present review demonstrate the efficacy of exercise programmes to improve hip flexion ROM, including active stretching [26] or rhythmic stabilizations [27].

As for exercise programmes aimed at improving trunk stability and core function, two studies were able to improve it [30,31]. This is consistent with the evidence available in other team sports [7]. These programmes not only help to improve trunk stability, but are also associated with a reduced risk of injury [7,10].

Regarding balance, the findings were mixed. Lopes et al. [32] found no significant improvements in static or dynamic balance, whereas Reis et al. [38] reported an improvement only in unilateral balance of the non-dominant limb. Although balance training has been associated with a lower incidence of ankle sprains in other sporting populations [52], the limited and inconsistent findings in futsal prevent firm conclusions regarding its effects or its relationship with injury prevention.

Regarding proprioception, Lopes et al. [32] found no significant changes in knee joint position sense, while Pérez-Silvestre et al. [36] reported significantly lower, and therefore better, AAE and VAE values in the intervention group than in the control group at the 10-week follow-up, although the significant within-group improvement was limited to VAE. Proprioceptive training has been associated with a reduction in ankle-sprain recurrence among previously injured athletes [53]; however, the studies included in this review did not directly examine whether improvements in proprioception were associated with fewer injuries. Therefore, these findings should be interpreted cautiously within the complex and multifactorial etiology of sports injuries [42].

The potential preventive effects of exercise-based programmes may be explained by several complementary mechanisms. Improvements in eccentric and isometric strength may help address strength deficits and imbalances associated with injury risk [11,46,47]. Improvements in power may enhance rapid force production and the capacity to absorb and redirect forces during high-intensity actions [44,45]. Proprioceptive, balance, and landing-mechanics training may improve neuromuscular control, postural stability, and joint alignment [40,44,52,52,53]. Greater flexibility and range of motion may also reduce movement restrictions and compensatory strategies, although isolated flexibility training is unlikely to be sufficient for injury prevention [9,48,49,51]. Differences in effectiveness between interventions may be related to their specificity, duration, training dose, and multimodal content. Programmes combining strength, plyometrics, balance, agility, and movement-control exercises may address several modifiable factors simultaneously, whereas more isolated interventions target a narrower outcome. Supervision, adherence, baseline characteristics, playing level, and the use of active rather than inactive comparators may also explain differences between studies. However, the limited number and heterogeneity of the available trials prevent firm conclusions regarding which programme is most effective.

Based on the available findings, multidimensional training programmes may have beneficial effects on injury outcomes; however, the evidence remains limited and of very low certainty [35] regarding improved performance [31,36,38,39,40] in futsal players. If future studies confirm a reduction in injury incidence, these programmes could help reduce the costs borne by teams, both at the professional level [3,6] and at lower levels [39,54], which is pertinent given the annual and progressive increase in sports injuries [6,55]. Moreover, they require very little equipment and may be cost-effective [3].

From a practical perspective, exercise-based programmes may be incorporated into regular training two to three times per week, commonly as 15–30 min warm-up sessions. The included interventions generally lasted between 4 and 12 weeks, although the available evidence does not establish a definitive minimum effective duration. Multicomponent programmes combining strength, plyometrics, balance, agility, and movement-control exercises appear to be a reasonable option because they address several modifiable factors simultaneously. However, the evidence does not support the superiority of any specific programme. Training load, exercise complexity, and supervision should be adapted to the age, experience, and competitive level of the players, as the limited number of studies prevented specific recommendations for youth versus adult or professional versus recreational populations.

Several limitations should be considered when interpreting these findings. First, the available evidence was based on a small number of studies, most with limited sample sizes. Only two studies assessed direct injury outcomes, and only one reported exposure-adjusted injury incidence. Differences in injury definitions and surveillance methods therefore prevented quantitative pooling of these outcomes. Clinical and methodological heterogeneity was also substantial, including variation in intervention content, comparator conditions, programme duration, participant characteristics, and outcome measures. Consequently, the pooled estimates should be interpreted as average effects across diverse comparisons rather than as the effect of a single, homogeneous intervention. This concern was particularly relevant for muscle strength, for which heterogeneity was considerable, and the pooled result was strongly influenced by individual studies. Although sensitivity analyses were conducted, the small number of studies precluded reliable subgroup analyses or meta-regression according to age, sex, playing level, intervention type, duration, or measurement method. For the same reason, publication-bias analyses had limited statistical power and should be interpreted cautiously. In addition, follow-up periods were generally short or inconsistently reported, several studies presented methodological limitations or concerns regarding risk of bias, and the evidence was derived predominantly from male players, limiting the generalizability of the findings. Finally, improvements in physical, neuromuscular, or biomechanical outcomes should not be interpreted as direct evidence of reduced injury incidence.

Future lines of research should focus on conducting RCTs with a more rigorous methodological design and long-term follow-up. It is essential that these studies not only assess injury incidence, but also aspects related to neuromuscular performance, to provide a more complete picture of the impact of exercise programmes on injury prevention in futsal.

## 5. Conclusions

Multidimensional exercise programmes may improve physical and neuromuscular outcomes in futsal players, showing positive effects on power, strength, and flexibility. However, direct evidence regarding injury prevention remains limited because only two reports assessed injury incidence. More high-quality research with long-term follow-up is needed to confirm these findings and further investigate their impact on injury incidence and neuromuscular performance.

## Figures and Tables

**Table 1:** Methodological quality assessment using the Physiotherapy Evidence Database (PEDro).

Author		Criteria										
	1 *	2	3	4	5	6	7	8	9	10	11	Score
Ayala et al. (2010) [26]	Yes	Yes	No	Yes	No	No	Yes	Yes	Yes	Yes	Yes	7
Bello et al. (2011) [27]	Yes	Yes	No	Yes	No	No	No	Yes	Yes	Yes	Yes	6
Bertolla et al. (2007) [28]	Yes	Yes	No	Yes	No	No	Yes	Yes	Yes	Yes	Yes	7
Gómez et al. (2023) [29]	Yes	Yes	Yes	Yes	Yes	No	Yes	Yes	Yes	Yes	Yes	9
Hamoongard et al. (2022) [40]	Yes	Yes	Yes	Yes	Yes	No	No	Yes	Yes	Yes	Yes	8
Jebavý et al. (2018) [30]	No	Yes	No	Yes	No	No	Yes	No	No	Yes	Yes	5
Jebavý et al. (2020) [31]	Yes	Yes	No	Yes	No	No	Yes	No	No	Yes	Yes	5
Lopes et al. (2019) [32]	Yes	Yes	Yes	No	No	No	No	No	No	Yes	Yes	4
Lopes et al. (2019) [33]	Yes	Yes	Yes	No	No	No	No	No	No	Yes	Yes	4
Lopes et al. (2020) [34]	Yes	Yes	Yes	No	No	No	No	No	No	Yes	Yes	4
Lopes et al. (2020) [35]	Yes	Yes	Yes	No	No	No	No	No	No	Yes	Yes	4
Pérez-Silvestre et al. (2019) [36]	Yes	Yes	Yes	Yes	Yes	No	Yes	Yes	Yes	Yes	Yes	9
Portilla-Dorado et al. (2019) [37]	Yes	Yes	No	No	No	No	No	Yes	Yes	Yes	Yes	5
Reis et al. (2013) [38]	Yes	Yes	No	Yes	No	No	No	Yes	Yes	Yes	Yes	6
Saryono et al. (2022) [39]	Yes	Yes	No	No	No	No	No	No	No	Yes	No	2

Criteria: (1) eligibility criteria were specified; (2) subjects were randomly allocated to groups; (3) allocation was concealed; (4) the groups were similar at baseline regarding the most important prognostic indicators; (5) there was blinding of all subjects; (6) there was blinding of all therapists who administered the therapy; (7) there was blinding of all assessors who measured at least one key outcome; (8) measures of at least one key outcome were obtained from more than 85% of the subjects initially allocated to groups; (9) all subjects for whom outcome measures were available received the treatment or control condition as allocated or, where this was not the case, data for at least one key outcome were analyzed by intention to treat; (10) the results of between-group statistical comparisons are reported for at least one key outcome; and (11) the study provides both point measures and measures of variability for at least one key outcome. (*) This criterion influences external validity, but not the internal or statistical validity of the trial. It has been included in the PEDro scale so that all items of the Delphi scale are represented on the PEDro scale. This item is not used to calculate the PEDro score.

**Table 2:** Risk-of-bias assessment using the RoB 2 tool of RCTs.

Intention to Treat Analysis	D1	D2	D3	D4	D5	Global
Ayala et al. (2010) [26]	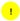	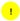	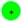	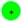	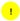	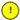
Bello et al. (2011) [27]	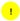	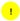	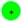	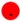	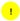	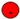
Bertolla et al. (2007) [28]	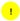	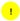	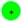	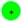	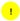	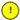
Gómez et al. (2023) [29]	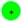	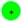	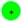	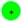	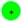	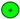
Hamoongard et al. (2022) [40]	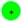	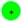	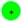	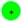	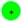	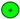
Jebavý et al. (2018) [30]	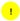	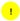	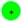	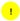	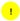	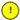
Jebavý et al. (2020) [31]	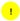	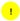	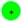	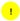	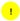	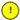
Pérez-Silvestre et al. (2019) [36]	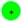	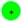	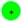	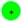	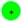	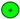
Portilla-Dorado et al. (2019) [37]	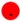	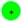	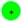	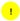	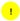	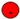
Reis et al. (2013) [38]	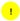	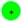	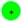	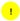	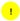	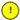
**Per-Protocol Analysis**	**D1**	**D2**	**D3**	**D4**	**D5**	**Global**
Lopes et al. (2019) [32]	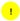	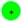	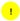	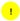	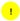	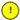
Lopes et al. (2) (2019) [33]	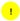	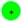	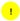	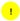	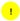	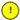
Lopes et al. (2020) [34]	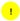	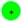	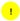	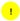	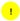	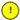
Lopes et al. (2) (2020) [35]	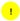	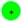	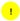	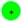	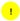	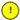
Saryono et al. (2022) [39]	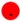	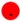	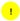	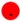	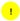	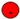

Domains: D1, Randomization process; D2, Deviations from intended interventions; D3, Missing outcome data; D4, Measurement of the outcome; D5, Selection of the reported result. Colors indicate risk-of-bias judgments: green, low risk of bias; yellow, some concerns; red, high risk of bias.

**Table 3:** Characteristics of included studies.

Report	Age (Years)	Sample (Level of Play, Intervention)	Duration (Weeks)	Outcome Measures	Results
Ayala et al. (2010) [26]	18–23	Professional players (Spanish First Division) G1: *n* = 10 (AS) G2: *n* = 8 (CG)	8 (+6 pre and 4 post)	ROM hip flexion (SLRT)	G1 showed significant improvements (26%) in ROM during SLRT after the intervention period. G2 did not demonstrate significant improvements in ROM in SLRT after the intervention. G1 achieved significant improvements compared to G2 in hip flexion ROM improvement. Four weeks later, ROM during SLRT decreased significantly in G1 compared to G2.
Bello et al. (2011) [27]	18–27	Professional players G1: *n* = 7 (RS) G2: *n* = 7 (PS)	4 months	Injury incidence, hip flexion ROM	G1 had fewer lesions compared to G2, although differences were not statistically significant. G1 significantly improved ROM in the dominant lower limb. No significant improvement in ROM for G1 in the non-dominant limb nor for G2 in the dominant or non-dominant limb. No significant differences between G1 and G2 in ROM.
Bertolla et al. (2007) [28]	17–20	Youth players G1: *n* = 6 (PM) G2: *n* = 5 (CG)	4 (+ post-evaluation)	Sit and reach test, trunk anterior flexion test	G1 improved significantly in both flexibility tests. These improvements were maintained for 15 days (non-significant decrease). G2 had no significant improvement in flexibility at the end of the intervention or at 15 days. G1 significantly better than G2 in improving flexibility, both acutely and chronically.
Gómez et al. (2023) [29]	18–24	Federated players G1: *n* = 11 (HIIT + NC) G2: *n* = 10 (HIIT)	4	30 × 15 IFT; CMJ; ABK jump; quadriceps and hamstring maximal isometric strength; isometric H:Q ratio	G1 and G2 significantly improved performance at 30 × 15 IFT. There were no changes for CMJ and ABK jump in G1 or G2. Neither G1 nor G2 demonstrated significant changes in quadriceps and hamstring isometric strength or H:Q ratio. Although G1 demonstrated greater improvements in most variables, they were not significant compared to G2.
Hamoongard et al. (2022) [40]	18–25	Non-professional players G1: *n* = 16 (IG) G2: *n* = 16 (CG)	8	Tuck jump test; mechanical landing during DJT	G1 significantly improved DKV in FI and FF, knee flexion in FI and FF, ankle dorsiflexion in FI and FF, and trunk flexion in FF. G2 did not demonstrate significant improvements in any variable. G1 showed significantly better differences than G2 in the variables DKV in IF and FF, knee flexion in IF and FF, dorsiflexion in IF and FF, and trunk flexion in FF. No significant differences between G1 and G2 in the variable’s trunk flexion in IF and lateral trunk flexion in IF and FF.
Jebavý et al. (2018) [30]	20–32	Professional players (Ukrainian First Division) G1: *n* = 10 (EG1) G2: *n* = 10 (EG2) G3: *n* = 10 (CG)	10	DSS function (score 1–5): diaphragm test, trunk flexion, back extension, hip flexion, IAP, side plank, lateral plank	G1 improved significantly in all tests except IAP. G2 improved significantly in all tests except hip flexion. G3 did not significantly improve in any test. G1 and G2 significantly superior to G3 in the IAP, lateral plank, and extension tests, while the hip flexion test was at the limit of significance. There was no significant difference between G1 and G2 for any test.
Jebavý et al. (2020) [31]	18–34	Professional players G1: *n* = 10 (IG) G2: *n* = 10 (CG)	10	DSS function (score 1–5): diaphragm test, trunk flexion, back extension, hip flexion, IAP, side plank, lateral plank	G1 showed significant improvements in all tests except hip flexion. G2 showed no significant changes in any of the tests. G1 showed significant changes in DSS function in the back extension, IAP, and lateral plank tests compared to G2 after the intervention.
Lopes et al. (2019) [32]	21–32	Non-professional players G1: *n* = 37 (IG) G2: *n* = 34 (CG)	10 (long-term, 10 weeks later)	Single-leg stance 30′; Y-balance test; proprioception dominant knee (JPS)	G1 showed no significant improvements in static and dynamic balance tests in the short or long term. No significant improvement in JPS of the dominant limb. G2 also showed no significant improvement in static and dynamic balance, nor in JPS. No significant differences between G1 and G2 in static and dynamic balance and JPS of the dominant knee.
Lopes et al. (2019b) [33]	21–32	Non-professional players G1: *n* = 37 (IG) G2: *n* = 34 (CG)	10 (long-term, 10 weeks later)	Agility T-test; SJ; sit and reach test; sprint 30 m	G1 no significant improvements in *t*-test, SJ, sit and reach, or 30 m sprint in short or long term. G2 no improvement in any of the parameters except SJ, although not significant. G1 no improvement in any of the performance parameters compared to G2 in the short or long term.
Lopes et al. (2020) [34]	21–32	Non-professional players G1: *n* = 37 (IG) G2: *n* = 34 (CG)	10 (long-term, 10 weeks later)	Isokinetic strength: peak concentric quadriceps and hamstring torque at 1.047 and 4.189 rad s^−1^; eccentric hamstring torque at 0.524 rad s^−1^; conventional and functional H:Q ratio; ankle-evertor latency time (ms)	G1 no significant difference in quadriceps and hamstring concentric peak torque at 1047 and 4189 rad s^−1^ compared to G2 in the short and long term. G2 significant decrease in quadricep concentric peak torque at 1047 rad s^−1^ compared to G1 in the long term. G1 short-term improvements in hamstring eccentric peak torque for both limbs in the short term compared to G2, not significant due to differences between groups. Significant differences in the long term. G1 significant improvements in conventional H:Q ratio of the dominant limb, G2 decreased values. Significant improvements in functional H:Q ratio in the dominant limb of G1 vs. G2. G1 improvements in short peroneal (non-dominant) and long peroneal (dominant) reaction time compared to G2 in the short term, not significant. No significant differences between groups in the long term.
Lopes et al. (2020) [35]	21–32	Non-professional players G1: *n* = 37 (IG) G2: *n* = 34 (CG)	20 (2 periods of 10 weeks separated by a period of 10 weeks)	Exposure time; recording and characteristics of injuries	G1 had more exposure time in training than G2. G1 sustained 24 injuries, while G2 reported 34 injuries. G2 demonstrated significantly more total injuries, acute injuries, MMII injuries, and training injuries than G1. G2 had a higher number of lost-time injuries and a higher number of days injured than G1. G2 higher percentage of ligament injuries (sprains) and injuries of medium severity compared to G1.
Pérez-Silvestre et al. (2019) [36]	16–24	Non-professional players G1: *n* = 17 (EG) G2: *n* = 17 (CG)	6 (long-term, follow-up at 10 weeks)	JPS dominant knee (AAE, RAE, VAE); CMJ	G1 showed non-significant reductions in AAE and RAE at 6 and 10 weeks and a significant reduction in VAE at 10 weeks, indicating improved proprioceptive consistency. No significant changes were observed in CMJ. G2 showed significant increases in AAE, RAE, and VAE at 10 weeks, indicating poorer proprioceptive performance, whereas no significant changes were observed at 6 weeks. No significant changes were observed in CMJ. At the 10-week follow-up, G1 showed significantly lower and therefore better AAE and VAE values than G2, whereas the between-group difference in RAE was not significant. No significant between-group differences were observed at 6 weeks. Neither group showed significant changes in CMJ.
Portilla-Dorado et al. (2019) [37]	16–32	National players G1: *n* = 8 (PNF) G2: *n* = 8 (FR) G3: *n* = 7 (CG)	8	Anthropometric measurements (BMI, sum of eight skinfolds, sum of MMII skinfolds, percentage of fat); jumping power (SJ, CMJ, ABK jump, unipodal jump); ST and BF activation with electromyography (RMS); sit and reach test; SLRT	G1 decrease in sum of eight folds. Furthermore, significant improvements in ABK jump and right single leg, no improvements in SJ and left single leg. G2 decrease in the sum of eight folds and MMII folds. Significant improvements in CMJ and ABK, without improvements in the SJ and single-leg jump. G3 cumulative decrease in MMII folds, significant decrease in CMJ, ABK, and right and left unileg jump. G1 and G2 improvements in activation of ST and right BF during SJ, and in flexibility, compared to G3. G1 better activation in right ST during CMJ, and better activation of right BF during single-leg hop, compared to G3. G2 better activation of the left ST during the SJ, and better activation of both ST and the right BF during the CMJ, compared to G3. For the “sit and reach” test, only G1 demonstrated significant improvements compared to G3.
Reis et al. (2013) [38]	16–18	Teenage players G1: *n* = 18 (IG) G2: *n* = 18 (CG)	12	Isokinetic strength: concentric quadriceps and hamstrings (60º/s, 240º/s), eccentric hamstrings (30º/s); conventional and functional H:Q ratio; *t*-test agility; sprint test (5m, 30m); technical skill (slalom dribbling); jump (CMJ, SJ); unilateral balance test	G1 significantly improved maximum concentric quadriceps and hamstring torque at 60º/s, and maximum eccentric hamstring torque in the dominant limb. In the non-dominant limb, improvements in maximum concentric quadriceps torque at 60º/s and in peak eccentric hamstring torque. G1 significant improvements in conventional H:Q (60º/s) and functional H:Q ratio (eccentric H 30º/s: concentric Q 240º/s). G1 significant improvements in *t*-test, 5 m and 30 m sprint, slalom, and CMJ. They also improved unilateral non-dominant limb balance. They did not significantly improve SJ. G2 no significant improvements for any parameter. G1 presented significant improvements compared to G2 for all performance tests, except for unilateral balance on the dominant limb.
Saryono et al. (2022) [39]	15–17	Young players G1: *n* = 53 (IG) G2: *n* = 42 (CG)	6	MMII isometric strength; ABD and ADD hip isometric strength	G1 showed significant improvements in isometric strength of LL and isometric strength of ABD and ADD. G2 significant improvements in isometric strength of MMII, significant decrease in isometric strength of ABD and ADD. G1 significantly better differences in isometric strength of ABD and ADD compared to G2. No significant differences between groups for LL isometric strength.

G1: Group 1; G2: Group 2; RS: Rhythmic stabilization; PS: Passive stretching; ROM: Range of motion; PM: Pilates method; CG: Control group; AS: Active stretching; SLRT: Straight leg raise test; IG: Intervention group; H:Q: Hamstrings–quadriceps; CMJ: Countermovement jump; SJ: Squat jump; EG1: Experimental Group 1; EG2: Experimental Group 2; DSS: Deep stabilization system; IAP: Intra-abdominal pressure; JPS: Joint position sensation; EG: Experimental group; AAE: Absolute angular error; RAE: Relative angular error; VAE: Variable angular error; PNF: Proprioceptive neuromuscular facilitation; FR: Foam roller; BMI: Body mass index; LL: Lower limbs; ABK: Abalakov; ST: Semitendinosus; BF: Biceps femoris; RMS: Root mean square; ms: Milliseconds; DJT: Down jump test; DKV: Dynamic knee valgus; IF: Initial phase; FF: Final phase; ABD: Abductors; ADD: Adductors; HIIT: High-intensity interval training; NC: Nordic curl; IFT: Intermittent fitness test.

## Data Availability

The authors confirm that we have all necessary permissions to reproduce material from other sources. Study Registration: CRD42024549885.

## Supplementary Materials

The following supporting information can be downloaded at: https://www.mdpi.com/article/10.3390/healthcare14162585/s1, Table S1: Search Strategy. Table S2: GRADE summary. Table S3: Characteristics of the intervention programmes. Figure S1. Meta-analysis of muscle power. Figure S2. Meta-analysis of muscle strength. Figure S3. Meta-analysis of muscle flexibility.
