# Supplementary material for: Effects of Exercise-Based Programmes on Injury Incidence and Physical and Neuromuscular Outcomes in Futsal Players: A Systematic Review and Meta-Analysis of Randomized Controlled Trials
Source: Healthcare (Basel). 2026 Aug 17;14(16):2585. doi: 10.3390/healthcare14162585 (PMC13512685; doi:10.3390/healthcare14162585)
Supplement: Supplementary file 1 [file healthcare-14-02585-s001.zip › healthcare-4462953-supplementary.pdf]

Supplementary Materials Table S1: Search Strategy.

**Pubmed**	("futsal" OR "indoor football" OR "Indoor soccer") AND ("Primary Prevention"[Mesh] OR "prevention") AND ("Exercise"[Mesh] OR "exercise" OR "Exercise Therapy"[Mesh] OR "training")
**Scopus**	TITLE-ABS-KEY ("futsal" OR "indoor football" OR "indoor soccer") AND TITLE-ABS-KEY ("Primary Prevention" OR "prevention") AND TITLE-ABS-KEY ("Exercise" OR "Exercise Therapy" OR " physical conditioning" OR "training")
**SPORTDiscus**	(DE "INDOOR soccer" OR "futsal" OR "Indoor football") AND (DE "PREVENTION" OR "prevention" OR DE "SPORTS injury prevention") AND (DE "EXERCISE" OR "exercise" OR DE "EXERCISE therapy" OR DE "PHYSICAL training & conditioning" OR "training")
**Web of Science**	TS=(("futsal" OR "indoor soccer" OR "indoor football") AND ("Primary Prevention"[Mesh] OR "prevention") AND ("Exercise"[Mesh] OR "exercise" OR "Exercise Therapy"[Mesh] OR "training"))
**PEDro**	futsal prevention<br>indoor soccer<br>indoor soccer prevention<br>indoor soccer exercise<br>indoor soccer exercise therapy<br>futsal exercise<br>futsal exercise therapy<br>futsal physical conditioning<br>futsal training

Supplementary Materials Table S2: GRADE summary

Outcome	Studies (participants)	Effect estimate	Risk of bias	Inconsistency	Indirectness	Imprecision	Publication bias	Certainty of evidence
**Direct injury outcomes**	2 (n = 85)	Lopes et al.: 6.5 vs. 11.6 injuries/1,000 player-hours; rate difference −5.1 (95% CI, −9.1 to −1.1). Bello et al.: 5 vs. 9 injury events; p = 0.096. No pooled estimate.	Serious[1]	Serious[2]	Not serious	Serious[3]	Undetected, but assessment was not reliable	**Very low** ⊕◯◯◯
**Muscle power**	5 (n = 185); 6 comparisons	Hedges' g = 0.616 (95% CI, 0.131 to 1.101)	Serious[1]	Serious[4]	Not serious	Not serious	Undetected, but assessment was underpowered	**Low** ⊕⊕◯◯
**Muscle strength**	4 (n = 223)	Hedges' g = 1.972 (95% CI, 0.214 to 3.731)	Serious[1]	Very serious[5]	Not serious	Serious[6]	Undetected, but assessment was highly uncertain	**Very low** ⊕◯◯◯
**Muscle flexibility**	3 (n = 105); 4 comparisons	Hedges' g = 0.569 (95% CI, 0.029 to 1.108)	Serious[1]	Not serious[7]	Not serious	Serious[8]	Possible, but not downgraded because of the very small number of studies	**Low** ⊕⊕◯◯

Explanatory notes

1. Downgraded because several contributing trials presented some concerns or high risk of bias.
2. Injury definitions, surveillance methods and denominators differed, and the two studies produced different statistical findings.
3. Downgraded because only two small studies were available and a comparable pooled effect could not be calculated.
4. Downgraded because heterogeneity was substantial (I^2 = 55.68%) and statistical significance was lost after excluding individual comparisons.
5. Downgraded by two levels because heterogeneity was considerable (I^2 = 95.64%) and the pooled estimate was strongly influenced by Reis et al.
6. Downgraded because the confidence interval was very wide and the sensitivity analyses included small and non-significant effects.
7. Statistical heterogeneity was low-to-moderate (I^2 = 35.95%) and was not statistically significant.
8. Downgraded because the confidence interval was close to the null effect and significance was lost after excluding several comparisons.

Supplementary Materials Table S3: Characteristics of the intervention programmes.

Report	Groups	Intervention program	Control intervention
**Ayala et al. (2010)**	G1: Active Stretching<br>G2: Control Group	Active stretching routine, 3 ss/w for 8 w on non-consecutive days, supervised by the trainer. 4 types of stretches per ss, 2 standing and 2 sitting. 1 repetition for unilateral, 2 for bilateral, maintaining stretching for 30 s (6x30s). Total stretching dose per ss was 180s, 20s rest between stretches.	They did not complete any stretching protocols.
**Bello et al. (2011)**	G1: Rhythmic Stabilization<br>G2: Passive Stretching	3 ss/w at the end of training for 4 months, performed by a physiotherapist. Player in supine position, where he performed 2 diagonal movements guided by the therapist, with maximum isometric contraction at the end of the range for 6 s, alternating between agonists and antagonists. 2 series of 10 stabilizations for each diagonal.	Passive stretching by a physiotherapist, 3 ss/w at the end of training for 4 months. 10s static stretch for thigh and leg muscles.
**Bertolla et al. (2007)**	G1: Pilates Method<br>G2: Control Group	3 ss/w for 4 w (total 12 ss, 25 min/ss), taught by a Pilates Method instructor. In ss 1-6 they performed protocol 1, consisting of the exs: "leg circles", "up and down", "scissor", "side kick", "the saw" and "spine stretch".<br>In ss 7-12 protocol 2, formed by "scissor", "shoulder bridge", "neckpull", "the saw", "spine stretch" and "push up".	Conventional team training routine.
**Gómez et al. (2023)**	G1: HIIT + Nordic Curl<br>G2: HIIT	1 ss/w for 4 w. The program was divided into several parts: (1) 10 min warm-up, running exs and active mobility. (2) HIIT 25 min, 8 exs with 40s of work and 15s of rest, for a total of 3 series. Ex: squat with vertical push (10kg bar), front plank with elbow flexion in pronation and supination of the forearm, squat with wall support with horizontal push (5kg), forward and backward sprint (5m), side plank, Jumping Jacks (5kg), lumbar rotations (5kg), and LL coordination exs with shoulders at 180º and external load (5kg). (3) Nordic Curl, 3 sets of 10 reps with 1 minute rest between sets. (4) Quadriceps, hamstrings, grastrocnemius and soleus stretches (10s per muscle group).	They completed the same program, one ss/w for 4 w, but without the Nordic Curl.

Supplementary Materials Table S3: Characteristics of the intervention programmes (continuation).

**Hamoongard et al. (2022)**	G1: Training + Dual Task<br>G2: Control Group	22 ss of neuromuscular training program for 8 w, 3 ss/w for the first 6 w, and 2 ss/w for w 7 and 8. Supervised by two trainers, focused on strength, agility, neuromuscular coordination, plyometrics, balance and proprioception. Examples: squats (bilateral and unilateral), lunges, drop jumps, horizontal jumps, single-leg positions on unstable bases, and single-leg CMJ. During execution, cognitive tasks with sensory stimuli, visual and auditory memory, motor learning or attention were included. 10 min submaximal warm-up on a stationary bike, 40 min of exercises (30-60s rest) and 5 min of cool down, total of 45-60 min.	Conventional futsal training.
**Jebavý et al. (2018)**	G1: Unstable Surfaces<br>G2: Stable surfaces<br>G3: Control Group	Experimental groups performed 25 ss for 10 w (2-3 ss/w), duration 30-45 min. Complex strength-resistance exercises with trunk musculature dominance from different postures (standing, hanging, sitting, lying down, kneeling). G1 performed the exs with unstable bases, while G2 used stable bases. G1 and G2 also performed 2 ss/w of 20 min with the entire team guided by a trainer to strengthen the trunk: "traditional" exercises (abs, lying squats and modifications).	Specific futsal exs (conventional training), along with the 2 ss/w of 20 min that the entire team carried out guided by the coach, with "traditional" exs.
**Jebavý et al. (2020)**	G1: Stabilization exs<br>G2: Traditional exs	25 ss of 30-40 min for 10 w (alternating 2 and 3 ss/w). Stabilization exs: inverted abdominals on a fitball and sliding board, one-handed planks, trunk rotations with external load on a fitball, one-legged squats on Bosu with an "Aqua bag". Tempo of 2/2/2/0 (exc/iso/con/rest phases between reps).	25 ss of 30-40 min for 10 w (alternating 2 and 3 ss/w). Traditional exs: abdominals, lateral crunch, trunk rotations, "deadbug". Tempo 1/0/1/0.
**Lopes et al. (2019a)**	G1: FIFA 11+<br>G2: Control Group	2 ss/w for 10 w of "FIFA 11+" during warm-up, administered by coach and 2 captains, 20 min. Parts: (1) easy running + dynamic stretches, (2) plyometrics, strength, agility and balance, and (3) sprints + changes of direction. They started at level 1, progressing to level 2 at 3 w, with 4 w at level 3.	Standard warm-up of 20 min. Combination of running, exs with a ball, static and dynamic stretching, progressing in intensity and speed.
**Lopes et al. (2019b)**	G1: FIFA 11+<br>G2: Control Group	2 ss/w for 10 w of "FIFA 11+" during warm-up, administered by coach and 2 captains, 20 min. Parts: (1) easy running + dynamic stretches, (2) plyometrics, strength, agility and balance, and (3) sprints + changes of direction. They started at level 1, progressing to level 2 at 3 w, with 4 w at level 3.	Standard warm-up of 20 min. Combination of running, exs with a ball, static and dynamic stretching, progressing in intensity and speed.
**Lopes et al. (2020a)**	G1: FIFA 11+<br>G2: Control Group	2 ss/w for 10 w of "FIFA 11+" during warm-up, administered by coach and 2 captains, 20 min. Parts: (1) easy running + dynamic stretches, (2) plyometrics, strength, agility and balance, and (3) sprints + changes of direction. They started at level 1, progressing to level 2 at 3 w, with 4 w at level 3.	Standard warm-up of 20 min. Combination of running, exs with a ball, static and dynamic stretching, progressing in intensity and speed.

Supplementary Materials Table S3 Characteristics of the intervention programmes (continuation).

**Lopes et al. (2020b)**	G1: FIFA 11+<br>G2: Control Group	2 ss/w for 20 w of "FIFA 11+" during warm-up, administered by coach and 2 captains, 20 min. Parts: (1) easy running + dynamic stretches, (2) plyometrics, strength, agility and balance, and (3) sprints + changes of direction. They started at level 1, progressing to level 2 at 3 w, with 4 w at level 3.	Standard warm-up of 20 min. Combination of running, exs with a ball, static and dynamic stretching, progressing in intensity and speed.
**Parsons et al. (2019)**	G1: FIFA 11+<br>G2: Control Group	"FIFA 11+" carried out 2 or 3 ss/w as a warm-up during the season (5 months), guided by coaches instructed by a physiotherapist. Duration of 20-30 min/ss Comprised of (1) easy running and dynamic stretching, (2) plyometrics, strength, agility and balance, and (3) sprints and changes of direction.	Standard warm-up, administered by the trainer, lasting approximately 10 min.
**Pérez-Silvestre et al. (2019)**	G1: Multi-Station Program<br>G2: Control Group	Multi-station program as a warm-up, 2 ss/w (6 w) guided by trainers or physical therapists, emphasis on strength, balance, plyometrics, stability, agility. 6 exs with 30s of rest between series and exercises. Examples: half squat (2x30s), one-leg half squat (2x10 rep/side), one-leg medicine ball throws (2x10 rep/side), CMJ (2x15 rep), Nordic curl (2x7 rep), 12m front/6m back run (2x3 rep).	Conventional futsal training routine.
**Portilla-Dorado et al. (2019)**	G1: PNF<br>G2: Foam Roller<br>G3: Control Group	G1 and G2 performed 3 ss/w in warm-up, for 8 w. PNF Group divided the weekly ss, 3 movements in each one. For each ex: 12s stretch, 12s contraction, and 12s stretch for each rep. 2 rep for each exs with 20s of rest between them.<br>Foam roller group performed 30s application per muscle group per leg, with 20s rest between applications and a total of 2 reps per group.	Control group did not receive any flexibility training.
**Reis et al. (2013)**	G1: FIFA 11+<br>G2: Control Group	"FIFA 11+" as a warm-up, 2 ss/w for 12 w. Composed of (1) low intensity running, stretching, and controlled contacts with a partner, (2) 6 series of strength, balance and jumping exercises, progressing with 3 levels of difficulty, and (3) sprint combined with changes of direction.	Standard warm-up: jogging and ball exs.
**Saryono et al. (2022)**	G1: "FIVE" Neuromuscular Warm-up<br>G2: Control Group	"FIVE" protocol in warm-up, 3 ss/w for 6 w, guided by coaches. Duration 15-20 min, 5 parts: (1) cardiovascular warm-up; (2) dynamic stretches; (3) strengthening (Examples: heel raises, Nordic curls or Copenhagen plank; (4) balance, plyometrics and agility; (5) preparation for ball play.	Regular futsal training.

G1: Group 1; G2: Group 2; ss: sessions; week: w; s: seconds; exs: exercises; rep: repetition; CMJ: Countermovement Jump; exc: excentric; iso: isometric; con: concentric; FIFA: Fédération Internationale de Football Association; PNF: Proprioceptive Neuromuscular Facilitation; FIVE: Futsal Injury Prevention and Enhance Performance; HIIT: High Intensity Interval Training; LL: Lower Limbs.

Study name	Hedges's g	Lower limit	Upper limit	p-Value
Gómez et al. (2023)	-0,261	-1,087	0,565	0,535
Lopes et al. (2019)	0,890	0,370	1,410	0,001
Pérez-Silvestre et al. (2019)	0,142	-0,515	0,799	0,672
Portilla-Dorado et al. (2019) G1	0,895	-0,269	2,059	0,132
Portilla-Dorado et al. (2019) G2	0,762	-0,493	2,016	0,234
Reis et al. (2013)	1,304	0,597	2,010	0,000
	0,616	0,131	1,101	0,013

Hedges's g and 95% CI

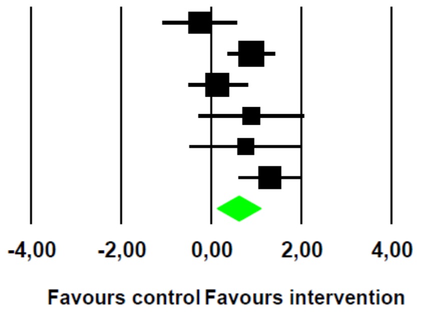

Supplementary Materials Figure S1**.** Meta-analysis of muscle power.

Study name	Hedges's g	Lower limit	Upper limit	p-Value
Gómez et al. (2023)	0,446	-0,387	1,279	0,294
Lopes et al. (2020)	0,594	0,074	1,114	0,025
Reis et al. (2013)	9,236	7,009	11,464	0,000
Saryono et al. (2022)	-0,315	-0,814	0,184	0,216
	1,972	0,214	3,731	0,028

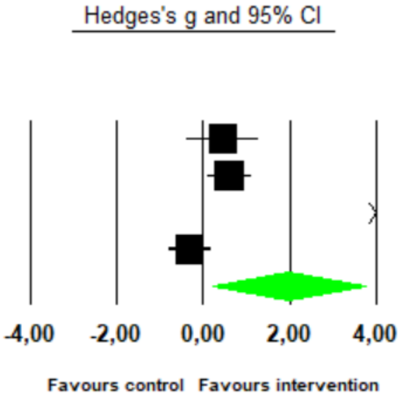

Supplementary Material Figure S2. Meta-analysis of muscle strength.

Study name	Statistics for each study			
	Hedges's g	Lower limit	Upper limit	p-Value
Bertolla et al. (2007)	0,603	-0,511	1,717	0,289
Lopes et al. (2019)	0,159	-0,337	0,656	0,529
Portilla-Dorado et al. (2019) G1	1,432	0,349	2,516	0,010
Portilla-Dorado et al. (2019) G2	0,636	-0,346	1,617	0,204
	0,569	0,029	1,108	0,039

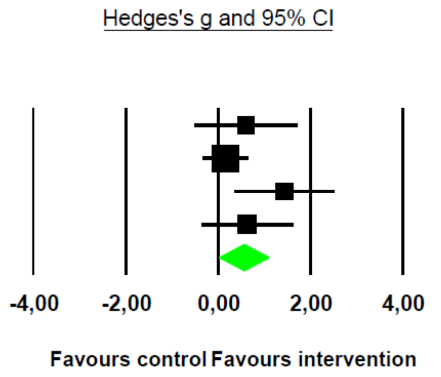

Supplementary Material Figure S3. Meta-analysis of muscle flexibility
